# Time, expectation and satisfaction: Patients’ experience at National Hospital Abuja, Nigeria

**DOI:** 10.4102/phcfm.v4i1.398

**Published:** 2012-07-19

**Authors:** Oluwagbenga Ogunfowokan, Muhammad Mora

**Affiliations:** 1 Department of Family Medicine, National Hospital Abuja, Nigeria

## Abstract

**Background:**

Long patient-clinic encounter time is typical of many hospital general outpatient departments (OPD) in Nigeria.

**Objectives:**

The objectives of our study were to determine the time spent by patients at the service points in the general OPD of the National Hospital Abuja (NHA), to establish the perception of patients regarding the patient–clinic encounter time, and to describe the level of satisfaction of patients with the services received.

**Methods:**

A cross-sectional study was conducted at the general OPD of the NHA. Information relating to the time spent at the various service points amongst others were obtained from 320 randomly selected patients using a patient administered validated questionnaire.

**Results:**

Two hundred and seventy (84.4%) patients responded adequately and were analysed. The median patient–clinic encounter time was 2.7 hours (range 0.2–7.2 hours). The long patient–clinic encounter time was accounted for mainly by the waiting time to see a doctor which was a median of 1 hour (range 0–5.6 hours) and time spent at the medical records with median of 0.5 hours (range 0–5 hours). There was a significant relationship between a short waiting time as perceived by patients, clinic visit encounters where patients’ expectations were met or surpassed, and overall patient satisfaction with the clinic visit encounter (*p* < 0.001).

**Conclusion:**

Reduction in patient–clinic encounter time and meeting patients’ pre-visit expectations could significantly improve patient satisfaction after clinic visit encounter at the general OPD of NHA.

## Abstrait

**Temps, attentes et satisfaction: L'expérience des patients à l'hôpital national d'Abuja, au Nigeria**

**Contexte:**

Les longues périodes de temps passées à la clinique par les patients sont caractéristiques des services de consultations externes (CE) générales de nombreux hôpitaux au Nigeria.

**Objectifs:**

Les objectifs de notre étude consistaient à déterminer le temps passé par les patients aux points de service dans les CE générales de l'Hôpital national d'Abuja (HNA), afin d’établir la perception des patients concernant le temps passé en clinique par les patients et de décrire le niveau de satisfaction des patients quant aux services reçus.

**Méthodes:**

Une étude transversale a été réalisée aux CE générales du HNA. Les informations en rapport avec le temps passé à divers points de service parmi d'autres ont été obtenues auprès de 320 patients sélectionnés de manière aléatoire en utilisant un questionnaire validé administré par les patients.

**Résultats:**

Deux-cent soixante-dix (84.4%) patients ont répondu de manière adéquate et ont été analysés. La moyenne de temps que les patients ont passé en clinique était de 2.7 heures (fourchette comprise entre 0.2 et 7.2 heures). Ce temps considérable passé en clinique s'expliquait principalement par le temps d'attente pour voir un docteur, qui était en moyenne d'une heure (fourchette comprise entre 0 et 5.6 heures) et le temps passé aux archives médicales avec une moyenne de 0,5 heures (fourchette comprise entre 0 et 5 heures). Il existait une relation importante entre un temps d'attente bref tel que perçu par les patients, les visites médicales au cours desquelles les attentes des patients étaient satisfaites ou surpassées et la satisfaction générale du patient quant à la visite médicale (*p* < 0.001).

**Conclusion:**

La réduction du temps passé par les patients en clinique et la satisfaction des attentes des patients avant la visite pourraient améliorer de manière significative la satisfaction du patient après la visite médicale aux CE du HNA.

## Introduction

### Key Focus

Satisfaction is an important outcome of healthcare. Clients judge the quality of care received based on their satisfaction with the services provided.

#### Background

Satisfaction during a health care encounter is related to the relationship between the patients’ expectations and experiences.^1^ Patients’ satisfaction can be improved when health workers meet their expectations and decrease the total time spent in a clinic.^1^ Experience with a healthcare service can have a direct impact on the patient's expectations of the services.^2^ Expectations refer to what patients think they will receive, what they desire, what they feel to be important or what they feel entitled to when seeking care.^3^ The relationship between expectations and experience is not always direct, but when experience deviates substantially from expectations, dissatisfaction results.^4^

Furthermore, patient satisfaction in terms of healthcare is important because it may influence patients’ attitudes towards healthcare services^5, 6^ Satisfied patients are more likely to seek medical advice, adhere to treatment recommendations, keep appointments, and refer other patients to their physician.^6, 7^

#### Trends

Waiting time at outpatient clinics in Nigeria has been found to be long^8–11^ which has resulted in a sense of dissatisfaction with the medical services provided in these clinics.^10, 11^ Lack of a time-specific appointment system has been suggested as a reason for this observation.^12^ Time-specific appointments are not the usual practice in our general outpatient clinics. Hence, most patients arrive at the general outpatient clinics within the same time block from 07:00 to 10:00 hours resulting in physicians being overwhelmed as well as long waiting periods before patient-physician contact can take place.

#### Objectives

No previous study has been conducted amongst family medicine general outpatient clinics of the National Hospital Abuja in terms of waiting periods or patient satisfaction even though there was a growing concern surrounding the improvement of care and patient satisfaction. The objectives of the study were to:

1. Determine the time spent by patients at the service points in the OPD of the family medicine department of National Hospital Abuja.
2. Know the perception of patients about the patient-clinic encounter time and
3. Describe the level of satisfaction of patients in terms of the services received.

#### Contribution to field

The study will add to the body of knowledge on patient waiting times and satisfaction with healthcare services in Nigeria where there is paucity of published work on the subject. It will also help in the formulation of a policy on an appointment system when patients are required to return to the clinic for follow-up visits after their initial visits are scheduled to come at a specific day and time or time block in an attempt to improve the quality of care, reduce waiting time and improve patient satisfaction.

## Ethical considerations

### Potential benefits and hazards

There was no potential hazard to the participants. Though the study did not directly benefit the participants, it had potential benefits to the society when the results are used to improve patient satisfaction and reduce waiting time.

### Recruitment procedures

Participation in the study was voluntary and patients were free to withdraw consent at any time. Withdrawal of consent would not cause a difference in the care received compared to those that did not withdraw consent.

### Informed consent

The study was explained to the patients and written informed consent was obtained prior to participation.

### Data protection

Data obtained from participants were kept confidential. Data were kept safe in a fire-proof cabinet; electronic versions were kept by the investigator.

## Methods

### Materials

All patients attending the general outpatient clinics of the family medicine department who had not previously participated in the pilot study were eligible for enrollment in the study.

### Setting

The family medicine department of National Hospital Abuja provides services at the general paediatric outpatient clinic (GPOPD), general adult outpatient clinic (GOPD) and HIV and/or AIDS outpatient clinic supported by the United State President's Emergency Plan for AIDS Relief (PEPFAR). National Hospital Abuja is located at the central district of Abuja, FCT which is located at the centre of Nigeria.

### Design

A cross-sectional study using a pre-tested, validated,^13^ patient administered^14^ questionnaire which was considered appropriate for the local conditions was carried out in the PEPFAR clinic, GOPD and GPOPD from 11 May - to 15 May 2009.

### Sample size

A confidence level of 95% and a power of 80% were used. The ratio of those having long waiting times compared to those with normal waiting times was 3:1 and at least 40% of those with long waiting times were dissatisfied (based on a pilot study that was conducted by the author using 20 patients in the same setting two months prior to this study). Using these estimates, a sample size of 248 patients was needed. Assuming a 20% non-respondent rate in this study, a minimum of 298 participants would be given the questionnaires. The participants in the pilot study were not part of this study. Epi info 3.4.3 was used for sample size calculation.

### Procedure

To avoid recall bias, consenting patients were informed about the importance of filling in the questionnaire as they move from the reception desk to other service points until the end of the patient-clinic encounter. A trained pre-designated observer also monitored the process to ensure accurate documentation. The first patient was selected by simple random sampling. Every consenting fifth patient was enrolled in the study until the estimated sample size was reached. Approximately 275 patients were usually seen daily in the clinics of the three units under the family medicine departments. Over the five days of data collection, about 1375 patients were estimated. The sample size was 298; this gives a sampling interval of approximately 5. Data captured in the questionnaire included the participants’ biodata, their time of arrival at the clinic, the time spent at reception, with the cashier, at the records department, with the nurse, waiting to see a physician, with the physician, and the total time of patient-clinic contact. Satisfaction with the various services received and the expectations and/or experiences of the participants were also elicited.

#### Analysing

Data were analysed with Epi Info 3.4.3 (CDC, Atlanta Georgia).^15^ Results are expressed as mean and standard deviation for continuous normally distributed variables and median with range for non-normally distributed continuous variables. Chi-square or Fisher's Exact test was used to find the relationship between categorical variables. Logistic regression was performed to examine the relationship between patient satisfaction and other independent variables. A *p*-value of 0.05 or less was considered statistically significant.

## Results

### Demography

Of the 320 patients who consented to fill in the questionnaire, 270 (84.4%) patients responded to all the questions. The participants included 146 (54.1%) women and 124 (45.9%) men. The mean age of participants was 37 years ± 10 (range, 14–76 years). One hundred and ninety five (72.1%) of the patients were married, 61 (22.7%) were single, 13 (4.8%) were widowed and 1 (0.4%) was divorced.

Seventy (25.9%; 95% C1 20.8 —1.6%) of the participants were new comers whilst 200 (74.1%; 95% C1 68.4 —79.2%) had attended the clinics previously. Most of the patients (141, 52.3%) came from Abuja whilst 80 (29.6%) came from the outskirt of Abuja, and the remaining 49 (18.1%) came from outside the Federal Capital Territory (FCT). One hundred and seventy two (63.6%) of the patients had tertiary education, 92 (34.1%) had below tertiary education, and 6 (2.3%) had no formal education. A median time of arrival in the clinics of 8:00 (5:00 – 2:00) was established.

#### Time spent by patients during clinic visit

**FIGURE 1:**
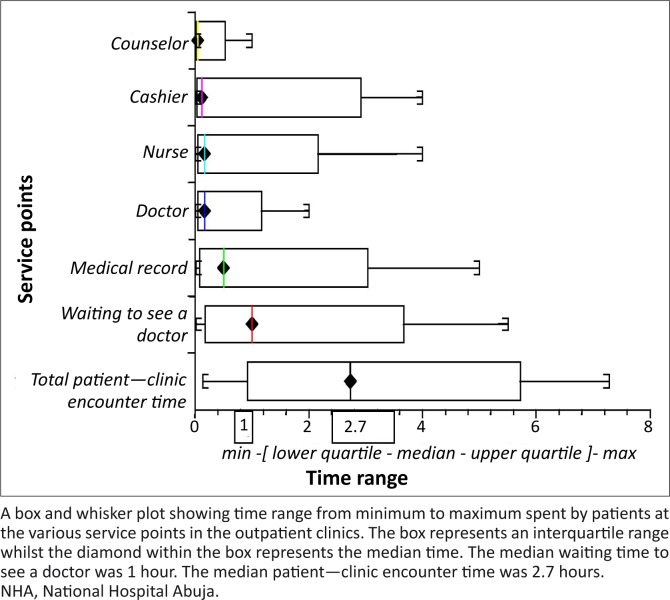
Time spent (hours) by patients during their clinic visit in the family medicine department of the NHA.

The median patient–clinic encounter time was 2.7 hours (range 10 minutes – 7.2 hours). This time was mainly due to the time spent waiting to see a doctor with a median of 1 hour (range 0–5.6 hours) (Figure 1).

#### Perceptions of patients about patient–clinic encounter time

**FIGURE 2:**
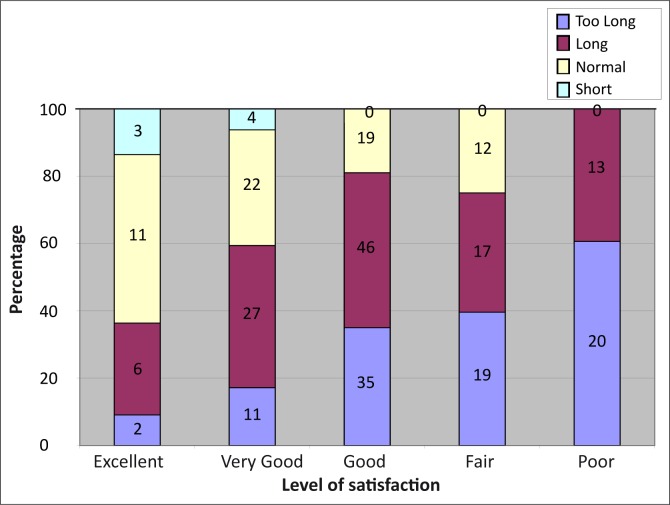
Patients’ level of satisfaction with overall services in relation to their perceptions about patient-clinic encounter time

Most of the participants (196, 72.6%) felt that the patient–clinic encounter time was either ‘long’ or ‘too long’ as shown in Table 1. The patients’ level of satisfaction with overall services decreased from ‘excellent’ to ‘poor’ as their perception of patient–clinic encounter time increased from ‘short’ to ‘too long’. All (33) of those who felt the services were poor also felt the time was either ‘long’ or ‘too long’ whilst 14 (63.6%) of those who felt the services were ‘excellent’ also felt the time was normal or short as shown in Figure 2.

**TABLE 1:** Perceptions of patients regarding patient–clinic encounter time in the family medicine department of National Hospital, Abuja.

Patients’ impression about patient–clinic encounter time	*f*	%	95% CI
Too long	87	32.2	27.0 - 38.6
Long	109	40.4	34.9 - 47.0
Normal	64	23.7	19.0 - 29.6
Short	7	2.6	1.1 - 5.3
No response	3	1.1	0.1 - 3.2

*f*, Frequency; CI, confidence interval.

#### Level of satisfaction of patients with services received

**FIGURE 3:**
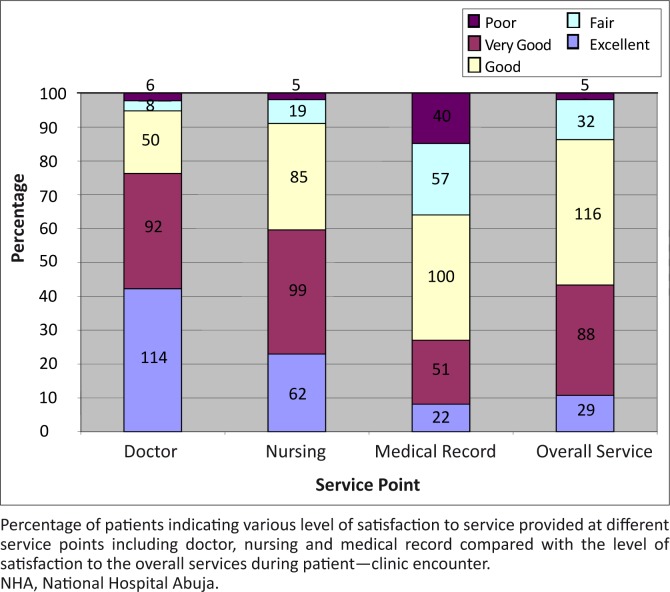
Level of satisfaction of patients with services received in the Clinics of Family Medicine department of NHA.

Participants were mostly satisfied with the services provided by their doctors. Two hundred and fifty six (94.8%) participants described their level of satisfaction with services rendered by the doctors as ‘excellent’, ‘very good’ or ‘good’ (Figure 3).

#### Expectations and/or experiences of the patients

**FIGURE 4:**
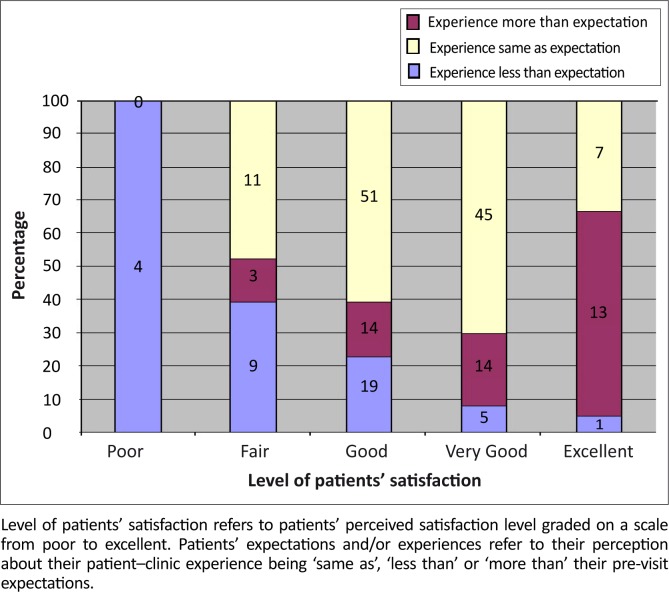
Patients’ level of satisfaction with overall services in relation to their expectations/experiences

Most of the patients 154 (57.2%) had experiences same as their expectations prior to their visit, 64 (23.5%) had experiences better than their expectations, and 52 (19.5%) had experiences worse than their expectations. All the patients who had experiences below their expectations prior to the visit felt the services were poor as shown in Figure 4.

#### Determinants of patients’ satisfaction

Patients with a satisfaction level ranging from ‘good’ to ‘excellent’ were classified as satisfied whilst those with a satisfaction level ranging from ‘poor’ to ‘fair’ were classified as dissatisfied. The relationship between satisfaction and other independent variables were determined using univariate analysis and then logistic regression analysis to remove the effect of confounders. A good impression about patient–clinic encounter time (normal or short visit time) was significantly associated with satisfaction (Fisher's exact Chi-square = 28; *p* < 0.001). Patients were also satisfied with the services when their clinic visit experiences were the same as or better than their expectations prior to their clinic visit (Fisher exact Chi-square = 49; *p* < 0.001).

## Discussion

The main aim of this study was to describe the satisfaction of patients attending the general outpatient clinics of the National Hospital Abuja with the services received and time spent during patient–clinic encounter. The findings will inform how to improve the efficiency, effectiveness and the quality of the services provided. We found that patients who had long waiting times were not satisfied with the services. This is similar to the findings by Sekandi et al. in Uganda in a study on a national hospital similar to NHA.^16^ Researchers in other settings found similar results.^17–19^

The range of waiting time in our study was 0–336 minutes. Those who did not wait at all (i.e. went straight from the nursing station where vital signs were taken to the doctor's office) experienced no waiting time. This disparity may be due to the few occasions when the doctor–patient encounter time was longer than usual. The range found in our study is much longer than 10–165 minutes found by Umar and associates in Sokoto, Nigeria. This difference may be due to the fact that our study was done in the general outpatient clinics whilst the study in Sokoto was done in Medicine, Surgery, Paediatrics, Obstetrics and Gynaecology clinics as well as general outpatient clinics. Appointment systems occurred in these non-general outpatient clinics; hence, the number of patients seen in the clinic was regulated. Long waiting time is common in most general outpatient clinics in Nigeria as was found by Ajayi in Ibadan, Nigeria, and Thatcher in Jos, Nigeria.^12, 20^ The median waiting time of 1 hour found in our study, however, contrasted with the finding by Christopher et al. and Camacho and co-workers who found a waiting time of 12 ± 11 minutes and 21 ± 15 minutes respectively.^21, 22^ This may be due to the use of time-specific appointment system in their settings. Appointment systems are not currently used in our general outpatient clinics.

Morrell et al. and Ridsdale and associates found a greater likelihood of patients feeling they had inadequate time with their physicians in visits scheduled to last 10 and 15 minutes respectively.^23, 24^ The participants in this study spent a median time of 9 minutes (2 minutes – 2 hours) in face-to-face encounters with the physicians. This is much lower than 24 ± 14 minutes in a study by Christopher and co-workers who found that spending less than 15 minutes with the patient was associated with patient dissatisfaction in terms of waiting time.^21^ However, our finding is similar to that of several studies that showed that ambulatory visits to family medicine physicians average between 9 and 18 minutes.^25–28^

Finally, we found that there is a significant relationship between patients’ overall satisfaction and meeting their expectations prior to the clinic visit. Jackson et al. noted that immediate post-visit satisfaction correlated with having no residual unmet expectations.^29^

Thompson and associates noted that patients were least satisfied when waiting times were longer than expected, relatively satisfied when waiting times were perceived as equal to expectations, and highly satisfied when waiting times were shorter than expected.^30^ Lin et al. also showed that significantly more patients rated their overall visit satisfaction at the highest level when their post visit estimate of time spent with the physician met or exceeded their pre-visit estimate of time needed.^14^

### Practical implications

This study has shown that patients attending the outpatient clinics of family medicine in National Hospital Abuja experience long waiting times contrary to their expectations. This resulted in a low level of patient satisfaction with overall services received. This result would be the basis for a change in the current practice where patients come without any form of appointment which resulted in overcrowded clinics.

### Limitation of the study

The main limitation of our study is its observational nature. The attitude of health workers to the patients, which was not examined in our study, could affect the level of satisfaction of the patients. Another limitation relates to the previous experiences of the subjects in clinic visit encounter which might bias their judgement during the clinic visit encounter in our study. We suggest future research that will incorporate healthcare workers’ attitude into the factors determining patient satisfaction.

### Recommendations

We recommend implementing an appointment system that is culturally sensitive to prevent overcrowding and long waiting times in the general outpatient clinics of National Hospital Abuja.

## Conclusion

Patient satisfaction is an important outcome which reflects the quality of health care of which patient waiting time is an important component.
